# Humanitarian surgical service utilization by a host country population: comparing surgery patterns between refugees and Tanzanians using an interrupted time-series analysis

**DOI:** 10.1186/s13031-021-00423-z

**Published:** 2021-11-22

**Authors:** Zachary Obinna Enumah, Sarah Rapaport, Hilary Ngude, Gayane Yenokyan, Amber Lekey, Peter J. Winch, Kent A. Stevens

**Affiliations:** 1 Johns Hopkins Global Surgery Initiative (JHGSI), Department of Surgery, Johns Hopkins Hospital, Tower 110 Doctor’s Lounge, 600 N. Wolfe Street, Baltimore, MD 21287 USA; 2 Department of International Health, Johns Hopkins School of Public Health, Baltimore, MD USA; 3 Tanzania Red Cross Society, Dar es Salaam, Tanzania; 4 Johns Hopkins Biostatistics Center, Department of Biostatistics, Johns Hopkins School of Public Health, Baltimore, USA; 5 Boston University School of Medicine, Boston, MA USA

## Abstract

**Background:**

While current estimates suggest that up to three million additional surgical procedures are needed to meet the needs of forcibly displaced populations, literature on surgical care for refugee or forced migrant populations has often focused on acute phase and war-related trauma or violence with insufficient attention to non-war related pathologies. To our knowledge, no study has compared refugee versus host population utilization of surgical services in a refugee camp-based hospital over such an extended period of twenty years. The aim of this paper is to first describe the patterns of surgical care by comparing refugee and host population utilization of surgical services in Nyarugusu refugee camp between 2000 and 2020, then evaluate the impact of a large influx of refugees in 2015 on refugee and host population utilization.

**Methods:**

The study was based on a retrospective review of surgical logbooks in Nyarugusu refugee camp (Kigoma, Tanzania) between 2000 and 2020. We utilized descriptive statistics and multiple group, interrupted time series methodology to assess baseline utilization of surgical services by a host population (Tanzanians) compared to refugees and trends in utilization before and after a large influx of Burundian refugees in 2015.

**Results:**

A total of 10,489 operations were performed in Nyarugusu refugee camp between 2000 and 2020. Refugees underwent the majority of procedures in this dataset (n = 7,767, 74.0%) versus Tanzanians (n = 2,722, 26.0%). The number of surgeries increased over time for both groups. The top five procedures for both groups included caesarean section, bilateral tubal ligation, herniorrhaphy, exploratory laparotomy and hysterectomy. In our time series model, refugees had 3.21 times the number of surgeries per quarter at baseline when compared to Tanzanians. The large influx of Burundian refugees in 2015 impacted surgical output significantly with a 38% decrease (IRR = 0.62, 95% CI 0.46–0.84) in surgeries in the Tanzanian group and a non-significant 20% increase in the refugee group (IRR = 1.20, 95% CI 0.99–1.46). The IRR for the difference-in-difference (ratio of ratios of post versus pre-intervention slopes between refugees and Tanzanians) was 1.04 (95% CI 1.00–1.07), and this result was significant (p=0.028).

**Conclusions:**

Surgical care in conflict and post-conflict settings is not limited to war or violence related trauma but instead includes a large burden of obstetrical and general surgical pathology. Host population utilization of surgical services in Nyarugusu camp accounted for over 25% of all surgeries performed, suggesting some host population benefit of the protracted refugee situation in western Tanzania. Host population utilization of surgical services was apparently different after a large influx of refugees from Burundi in 2015.

## Background

Surgical care is a cornerstone of healthcare globally, yet the literature on surgical output, capacity, and burden of disease in conflict settings is limited. While current estimates suggest that up to three million additional procedures are needed to meet the needs of forcibly displaced populations, literature on surgical care for refugee or forced migrant populations has often focused on acute phase and war-related trauma or violence with insufficient attention to non-war related pathologies among these populations [1–4]. Only a handful of studies investigate non-war related surgical output, especially in a protracted refugee or conflict setting [4–6].

The United Nations High Commissioner for Refugees (UNHCR) estimated 79.5 million forcibly displaced persons at the end of 2019, with approximately 26 million being refugees [7]. Tanzania has hosted refugees for over six decades, and it currently is home to approximately 242,171 refugees divided amongst three camps—Nyarugusu, Mtendeli and Nduta [8]. The burden of surgical disease and surgical capacity in refugee camp settings, such as in Tanzania, is poorly understood. While broadly speaking, displaced populations reside in a variety of settings (e.g. refugee camps, local integration, or resettlement), for refugees in Tanzania, camps are the most common habitation as refugees are required by law to live in designated camps [9–12]. In 2015, Tanzania received a large influx of refugees from Burundi, prompting them to reopen previous refugee camps and simultaneously doubling the population of its largest camp, Nyarugusu refugee camp.

Nyarugusu camp is located in Kigoma region, Tanzania, which has a long land border with Burundi to its northwest. Created in 1996, the population of Nyarugusu camp is approximately 150,000 individuals. Refugees come primarily from the Democratic Republic of Congo (DRC) and Burundi. As of 2018, the camp is divided into 12 administrative zones and 142 villages [13]. Since 2004, the Tanzanian Red Cross Society (TRCS) coordinates medical services within Nyarugusu camp [14, 15]. The camp supports one main dispensary hospital, multiple health centers, and health posts that serve refugees and local Tanzanians who live in the surrounding area. The hospital currently has two major operating theatres and one minor operating theatre. Anesthesia available includes general anesthesia, spinal anesthesia, and local anesthesia; however, there is currently no functioning universal anesthesia machine to permit intubation. The total catchment population for Nyarugusu is estimated to be 200,000 individuals. Camp-based services are provided free of cost in contrast to local and district hospitals that charge a fee for both refugees and Tanzanian nationals accessing care [16]. Tanzanians are able to access the health facility in the refugee camp free of charge. For all patients, cases requiring complex surgical management are referred to other centers within Kigoma region (e.g., Kabanga Hospital, Maweni Regional Referral Center) or tertiary hospitals outside of Kigoma region (e.g., Bugando Medical Centre) guided by UNHCR principles and other local operating procedures [17]. However, a significant amount of surgery for basic obstetrical and general surgical care is provided in the camp hospital.

Some studies have explored host community utilization of health services in humanitarian settings [18–20] but to our knowledge, no study has specifically examined host population utilization of surgical services in a refugee camp-based hospital. The aim of this paper is to first describe the patterns of surgical care by comparing refugee and host population utilization of surgical services in Nyarugusu refugee camp between 2000 and 2020. Secondly, we hypothesized that a large influx of Burundian refugees in 2015 differentially affected the proportion and number of surgical cases among refugees and Tanzanians.

## Methods

We retrospectively reviewed surgical logbooks for major procedures that are maintained by camp staff working with the Tanzania Red Cross Society. Using descriptive statistics and time series analysis, we sought to explore host and refugee utilization of surgical services in a refugee camp-based hospital.

### Data collection

Photos of logbooks recording major operations (defined below) were taken and digitized into a standardized Microsoft Excel form. Data abstracted included operation date (separately abstracted as day, month, year), time of operation, age of patient, sex, nationality, indication, procedure, post-operative diagnosis, and anesthesia used. Major procedures were defined as invasive operative procedures that typically involve extensive resection (e.g. entering a body cavity, removing an organ, etc.). These procedures include, but are not limited to, caesarean section, herniorrhaphy, hemorrhoidectomy, laparotomy, amputation, bilateral tubal ligation, total abdominal hysterectomy, appendectomy, thyroidectomy, and hydrocelectomy. Procedures were included if nationality data was available and excluded if nationality data was missing or illegible. Patients with nationality listed as dual (e.g. Democratic Republic of Congo/Tanzanian) or Kenyan were said to be missing.

### Statistical analysis

Data were stratified into two groups (refugee versus host population) and patient characteristics were compared between the two groups. Top indications for surgery and type of procedures were also compared between the two groups. Categorical variables were reported as whole numbers (n) with associated percentages and compared using Chi-square analysis or Fischer exact test.

We examined trends in number of surgeries over time by refugee or host population status presented as scatter plots. Tens of thousands of Burundian refugees arrived in Tanzania beginning in April 2015. To assess the effect of a large influx of refugees in 2015 on surgical output and refugee versus host population status, we performed a multiple group, interrupted time series analysis with January 2015 to March 2015 as the “intervention period” (see Interrupted Time Series Analysis below) since refugees began arriving in April 2015. Data were divided into quarters.

Interrupted Time Series Analysis (ITSA) is a quasi-experimental design that allows researchers to study the effect of a particular health care intervention or event on an actual or predicted outcome [21, 22]. We used this method to assess the impact of a mass influx of refugees on surgical output for refugee and host populations (Tanzanians).

Repeated observations (in this case, counts of surgery by quarter) are recorded over time. In simplest of approaches (and in this case), a single intervention is studied producing two time periods—one before the intervention, one after. Intervention in this context refers to the influx of Burundian refugees beginning in April 2015. Rates (or counts) of surgery are compared before and after the intervention, as well as rate of change (slope), by utilizing segmented regression. Given our data was “count” data and our variance was greater than the mean, we utilized Poisson distribution in our population average marginal model estimated using generalized estimating equations (GEE) and accounted for overdispersion in our model. The model included first-order autoregressive working correlation structure.

The final model is represented by the following,

$$\upmu t=\upbeta 0+\upbeta 1\mathrm{Tt}+\upbeta 2\mathrm{Z}+\upbeta 3\mathrm{ZTt}+\upbeta 4\mathrm{Xt}+\upbeta 5\mathrm{XtT}+\upbeta 6\mathrm{ZXt}+\upbeta 7\mathrm{ZXtTt}$$

where μ represents the outcome of interest, the average surgery count, T represents time (measured in 3-month quarters), X is a dummy variable representing the intervention period (post-intervention = 1, otherwise 0), Z is a dummy variable representing group status (refugee = 1, otherwise 0), X_t_T_t_, ZT_t_, ZX_t_, and ZX_t_T_t_ are all interaction terms of the above variables. By utilizing a reference group, we partly accounted for other local, contextual and secular trends that may have influenced case numbers, such as an earthquake that damaged the original operating theatre in 2017 (as this would affect both groups). All data analysis was performed with Stata 16 (StataCorp. 2019. *Stata Statistical Software: Release 16*. College Station, TX: StataCorp LLC.).

### Ethical clearance

Ethical approval was obtained from the Johns Hopkins Medical Institutions Institutional Review Board and Tanzanian Commission on Science and Technology.

## Results

### Characteristics of study population

Between December 2000 and September 2020, a total of 10,489 operations were performed in Nyarugusu refugee camp after excluding those not meeting inclusion criteria. Refugees underwent the majority of procedures in this dataset (n = 7,767, 74.0%) versus Tanzanians (n = 2,722, 26.0%). Of those included in the analysis, 9,516 were female (91.0%) and 946 were male (9.0%). The majority of procedures were performed on individuals aged 18 to 29 (n = 5,951, 56.7%) followed by individuals aged 30 to 44 (n = 2,623, 25.0%). Regarding nationality, individuals from the Democratic Republic of Congo comprised over half of the observations (n = 5,898, 56.2%), followed by Tanzanians (n = 2,722, 26.0%), Burundians (n = 1,714, 16.3%) and Other (n = 155, 1.5%) which accounted for individuals classified as Rwandan or refugee. The most common operation performed was caesarean section (n = 8,267, 79.4%), followed by bilateral tubal ligation (n = 725, 7.0%), herniorrhaphy (n = 713, 6.8%), and exploratory laparotomy (n = 539, 5.2%).

Demographic and procedural frequencies were compared across refugee versus host population (Tanzanians). Refugees were more likely to be female (n = 7,075, 91.3%) compared to Tanzanians (n = 2,441, 89.9%, p = 0.022). Age distribution between the two groups (refugee versus Tanzanian) was significantly different (p < 0.001) (Table 1). Similarly, yearly breakdown of operations demonstrated heterogeneity with 2019 being the calendar year with highest number of operations (total n = 965; refugees: n = 706, Tanzanian, n = 259) and 2001 being the full calendar year with lowest number of operations (total n = 202; refugees, n = 167, Tanzanians, n = 35) (Table 1).

**Table 1:** Demographic and ecological characteristics of surgical patients

Variable	Tanzanian patients	Refugees	*p* value
N	2722	7767	
Age category			< 0.001
Under 5	31 (1.1%)	79 (1.0%)	
Age 5 to 17	273 (10.0%)	819 (10.5%)	
Age 18 to 29	1629 (59.8%)	4322 (55.6%)	
Age 30 to 44	597 (21.9%)	2026 (26.1%)	
Age 45 to 60	78 (2.9%)	252 (3.2%)	
Age 60 +	69 (2.5%)	145 (1.9%)	
Missing	45 (1.7%)	124 (1.6%)	
Female sex	2441 (89.9%)	7075 (91.3%)	0.022
Nationality			< 0.001
DRC	–	5898 (75.9%)	
Burundi	–	1714 (22.1%)	
Tanzanian	2722 (100.0%)	0 (0.0%)	
Other*	–	155 (2.0%)	
Year			< 0.001
2000	0 (0.0%)	6 (0.1%)	
2001	35 (1.3%)	167 (2.2%)	
2002	53 (1.9%)	267 (3.4%)	
2003	51 (1.9%)	206 (2.7%)	
2004	85 (3.1%)	270 (3.5%)	
2005	62 (2.3%)	320 (4.1%)	
2006	68 (2.5%)	276 (3.6%)	
2007	61 (2.2%)	235 (3.0%)	
2008	81 (3.0%)	230 (3.0%)	
2009	119 (4.4%)	258 (3.3%)	
2010	133 (4.9%)	338 (4.4%)	
2011	101 (3.7%)	346 (4.5%)	
2012	175 (6.4%)	391 (5.0%)	
2013	220 (8.1%)	417 (5.4%)	
2014	267 (9.8%)	457 (5.9%)	
2015	218 (8.0%)	607 (7.8%)	
2016	170 (6.3%)	622 (8.0%)	
2017	224 (8.2%)	581 (7.5%)	
2018	188 (6.9%)	633 (8.2%)	
2019	259 (9.5%)	706 (9.1%)	
2020	149 (5.5%)	423 (5.5%)	

^*^Other includes individuals classified as Rwandan or broadly as “refugee’

### Patterns of utilization in host versus refugee population

Regarding frequency of most common procedures performed in the camp, there was no statistically significant difference between relative frequency of caesarean sections in Tanzanians versus refugees (TZ: n = 2119, 78.5%; REF: n = 6148, 79.7%, p = 0.18), but caesarean sections were the most common procedure for both Tanzanians and refugees. Bilateral tubal ligations were more common in Tanzanians (n = 215, 8.0%) versus refugees (n = 510, 6.6%, p = 0.018). Herniorrhaphies were more common in Tanzanians (n = 211, 7.8%) when compared to refugees (n = 502, 6.5%, p = 0.021). Finally, there was no statistically significant difference between relative frequencies of exploratory laparotomies when comparing Tanzanians (n = 133, 4.9%) and refugees (n = 406, 5.3%, p = 0.50). Statistically significant differences between host and refugee population were found in the following operations: appendectomy, orchidectomy, ventral suspension, hemorrhoidectomy, and lipoma excision. Additional procedures are presented in Table 2.

**Table 2:** Top procedures performed in host versus refugee population

Operation type	Tanzanian	Refugee	*p* value
Caesarean section	2119 (78.5%)	6148 (79.7%)	0.18
Bilateral tubal ligation	215 (8.0%)	510 (6.6%)	0.018
Herniorrhaphy	211 (7.8%)	502 (6.5%)	0.021
Exploratory laparotomy	133 (4.9%)	406 (5.3%)	0.50
Hysterectomy	67 (2.5%)	144 (1.9%)	0.051
Hydrocelectomy	30 (1.1%)	91 (1.2%)	0.77
Appendectomy	20 (0.7%)	119 (1.5%)	0.002
Orchidectomy	17 (0.6%)	23 (0.3%)	0.017
Repair	25 (0.9%)	101 (1.3%)	0.12
Oophorectomy	2 (0.1%)	22 (0.3%)	0.060*
Ventral suspension	2 (0.1%)	27 (0.3%)	0.018*
Cystectomy	7 (0.3%)	30 (0.4%)	0.33
Evacuation	9 (0.3%)	22 (0.3%)	0.69
Excision	9 (0.3%)	18 (0.2%)	0.38
Colporraphy	3 (0.1%)	16 (0.2%)	0.43*
Haemorrhoidectomy	13 (0.5%)	8 (0.1%)	< 0.001
Myomectomy	3 (0.1%)	14 (0.2%)	0.58*
Hysterotomy	8 (0.3%)	10 (0.1%)	0.073
Lipoma Excision	8 (0.3%)	8 (0.1%)	0.028

^*^Fisher exact test performed given cell with less than count of five (5)

### Time trends in patterns of utilization in host versus refugee population

**Fig. 1:**
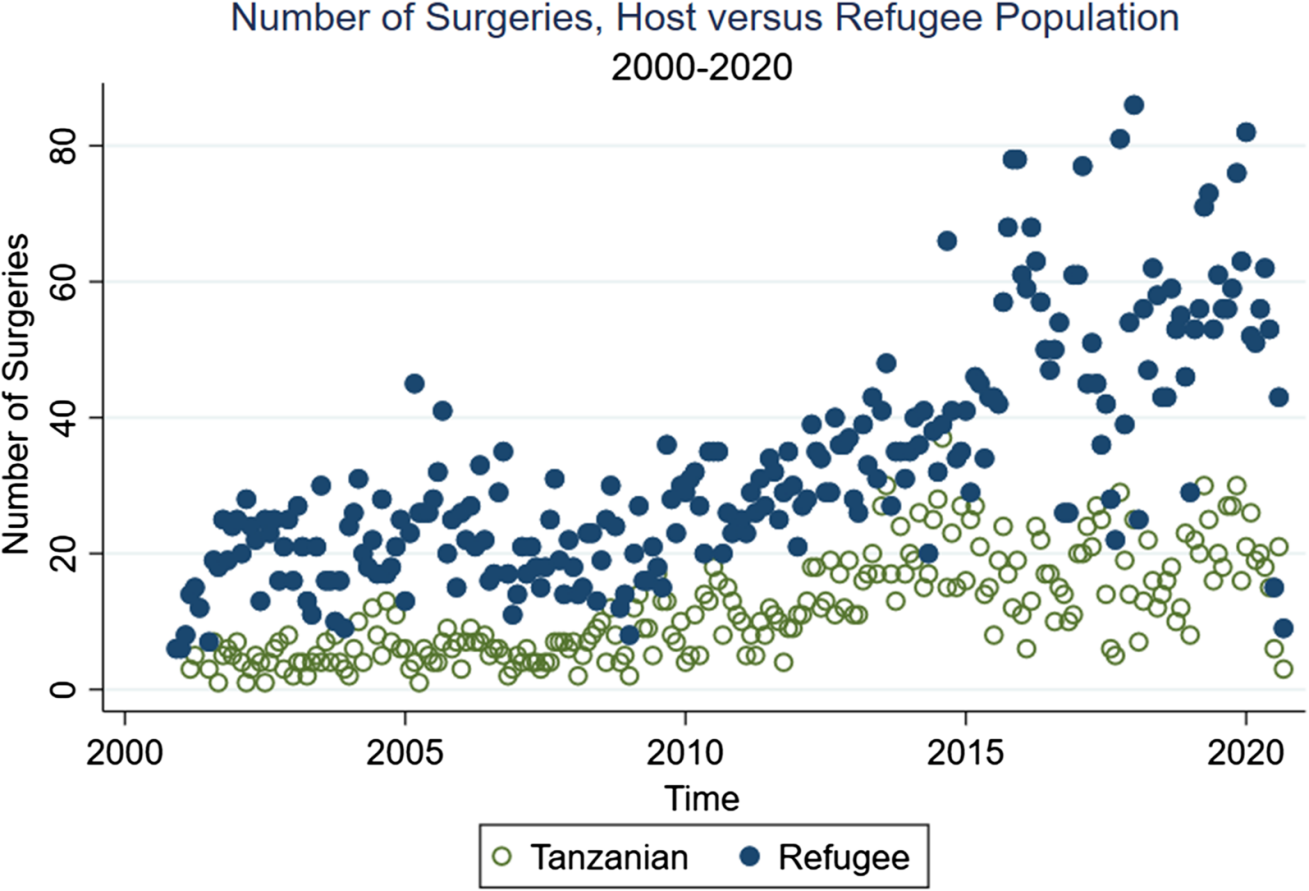
Scatter plot of number of surgeries among host versus refugee population, 2000–2020

Both groups exhibited a general increasing trend in utilization of surgical services in Nyarugusu camp. The year with highest count of surgeries for Tanzanians and refugees was 2019 (Tanzanians: n = 259, Refugee; n = 706). Data for 2020 only included up through September of that year. Surgery counts over time by group are displayed in Fig. 1.

### Interrupted time series analysis

**Fig. 2:**
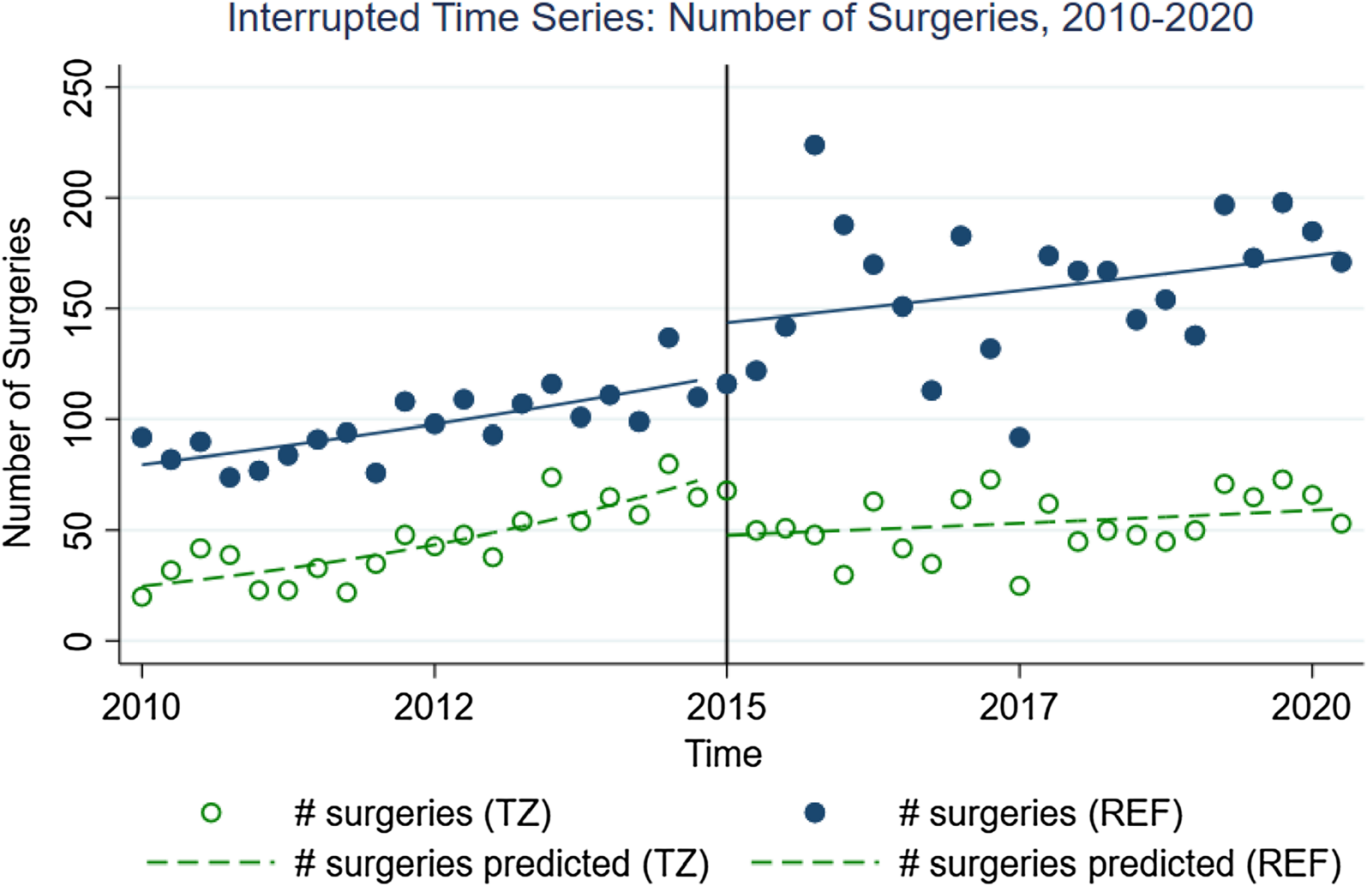
Time Series Analysis for all surgeries, refugees (REF) versus Tanzanians (TZ), 2010–2020 with single interruption in 2015 representing influx of Burundian Refugees

Baseline characteristics were compared between Tanzanians and refugees in quarter 1 of 2010 (start time of time series analysis). No significant differences in age or sex were found. We then compared both incidence rate ratios and change in rate of incidence rate ratios between refugees and host population in the pre- and post-2015 periods and results of the Poisson time series model are available in Table 3. Regarding all surgeries performed, the baseline average number of surgeries for Tanzanians was 24.8 surgeries in the first quarter (95% CI 19.1–32.4). There was a mild but significant increase among Tanzanians in the average number of surgeries of 5.8% per quarter up until 2015 (95% CI 3.6–8.0% increase). In the first quarter, refugees had 3.21 times higher average number of surgeries compared to Tanzanians (95% CI 2.35–4.38). The baseline average number of surgeries for refugees was 79.7 surgeries in the first quarter (95% CI 67.9–93.7). For refugees, there was also a significant increase in the average number of surgeries of 2.1% per quarter until 2015 (95% CI 0.7–3.5% increase), with statistically significant differences in this time trend between groups in the pre-2015 period (p = 0.005). See Fig. 2.

**Table 3:** Interrupted time series model output

	IRR	95% confidence interval	*p* value
**Intercept:** baseline average number of surgeries among Tanzanians, 2010, Q1	24.8	19.1–32.4	< 0.001
**T,** slope prior to intervention among Tanzanians (ratio of average number of surgeries per quarter)	1.06	1.04–1.08	< 0.001
**Z,** ratio of average surgery counts between Refugees and Tanzanians at baseline (2010, Q1)	3.21	2.35–4.38	< 0.001
**Z x T,** ratio of slopes prior to intervention between Refugees and Tanzanians	0.96	0.94–0.99	0.005
**X,** ratio of average surgery counts in the period immediately following intervention (2015, Q1) to the last pre-intervention quarter (2014, Q4) among Tanzanians	0.62	0.46–0.84	0.002
**X x T,** ratio of postintervention and pre-intervention slopes for Tanzanians	0.96	0.93–0.98	0.001
**Z x X interaction,** ratio of the post-intervention change in average number of surgeries between Refugees and Tanzanians, in the period immediately following intervention (i.e. 2015, Q1) compared to the last pre-intervention quarter (2014, Q4)	1.92	1.35–2.74	< 0.001
**Z x X x T interaction,** ratio of ratios of post-vs. pre-intervention slopes between Refugees and Tanzanians (“difference in difference” on multiplicative scale)	1.04	1.00–1.07	0.028

For all surgeries and immediately following the influx of Burundian refugees in 2015, there was a significant 38% decrease in the average number of surgeries among Tanzanians (IRR = 0.62, 95% CI 0.46–0.84) and a non-significant 20% increase among refugees (IRR = 1.20, 95% CI 0.99–1.46). The new baseline (1st quarter of 2015) number of surgeries among Tanzanians immediately following the influx was 47.8 surgeries (95% CI 39.0–58.6). The new baseline average number of surgeries among refugees immediately following the influx was 143.8 surgeries (95% CI 127.9–161.7). There was a non-significant 1.1% increase in the number of surgeries over time for Tanzanians and a 1.0% increase per quarter among refugees in the post-2015 period (IRR = 1.01, 95% CI 1.00–1.02), with no statistically significant differences in post-2015 time trend between these two groups (p = 0.915). Finally, the IRR for the difference-in-difference (ratio of ratios of post versus pre-intervention slopes between refugees and Tanzanians) was 1.04 (95% CI 1.00–1.07), and this result was significant (p=0.028). See Table 3 and Fig. 2.

## Discussion

We sought to understand the differences and patterns of refugee camp surgical services utilization by both a refugee population and host population (Tanzanians). The strengths of this study include (1) its use of a large dataset over a 20-year period in one of the world’s largest refugee camps; (2) incorporating a quasi-experimental method to better understand trends in surgical service utilization among two distinct populations; and (3) generating new knowledge regarding host population utilization of surgical services compared to refugee utilization in a refugee camp based hospital. In considering the study performed, we believe its findings highlight several key issues in understanding and contextualizing surgery in the context of conflict settings.

Firstly, scholars have suggested that when large influxes of refugees occur, there is a type of “humanitarian spill over” that can both positively and negatively affect host communities [23, 24]. Our study supports this theory given the significant utilization of surgical services in Nyarugusu refugee camp by Tanzanians. Tanzanians may enter the refugee camp to seek treatment free of charge, despite changing refugee policies in Tanzania that have restricted the freedom of movement of refugees. This finding implies that Tanzanian patients are also benefiting from the humanitarian efforts and health landscape, similar to how others have discussed host populations benefitting or utilizing refugee based health facilities [20].

Secondly and relatedly, there appeared to be a change in surgical output in the refugee camp following the influx of Burundian refugees in 2015. Not only did the relative level and rate of surgery in the camp increase for refugees following the influx (as well as a statistically significant difference in difference), but there was also a concomitant decrease of 38% among Tanzanians. Further, the rate of change of surgeries occurring among this group decreased by almost 4% and appeared to level off when compared to the pre-2015 period. One possible explanation for this change might be that capacity in Nyarugusu was reached. Initially designed to only accommodate 50,000 individuals, such a large and immediate change to the population dynamics could have caused Tanzanians to access the facilities relatively less frequently [25–27]. These findings must be understood, though, in the context that estimates of underlying population per month or quarter among the study population was not available, so it does not account for underlying changes to sub-populations.

Thirdly, this study supports the findings of several others that in conflict zones, a significant proportion of surgical output is not necessarily for trauma or violence related injuries but rather mirrors the surgical burden of non-conflict settings [4, 5, 28]. The large proportion of Tanzanian patients treated in a conflict/post-conflict zone hospital (i.e. Nyarugusu refugee camp dispensary) corroborates this theory even further: not only is non-trauma related surgery occurring in this context, but host populations are utilizing health services in a similar pattern to other non-conflict settings. In addition, relative utilization of surgical services between Tanzanians and refugees were quite similar with reference to the top five procedures performed in the camp (caesarean section, bilateral tubal ligation, herniorrhaphy, exploratory laparotomy and hysterectomy). This again emphasizes that of other studies that surgery in conflict or post-conflict populations may mirror that of the general population [6, 29]. Further, caesarean sections were the most common procedure for both Tanzanians and refugees, which is in line with other studies in Tanzania demonstrating high surgical output of caesarean sections [30].

Finally, forced migrants and conflict and post-conflict settings are often excluded from global surgery research and estimates [31]. Given the significant surgical output in this refugee context for both refugees and host populations, we suggest that this type of data be more formally included in these types of studies, including how host populations use camp-based facilities. As we assess whether or not certain indicators are being met with regard to global surgery (e.g. 5000 procedures per 100,000 by year 2030), it will be imperative to include populations of forced migrants in these estimates [32]. Additional research might include prospective surgical registries that captures both refugee and host population procedures and outcomes, as well as serial cross-sectional studies to assess changing prevalence of surgical disease among refugee and host populations using household surveys [33, 34]. Such estimates will be vital in humanitarian planning and programming.

Our study is not without limitations. While interrupted time series analysis can be used as a quasi-experimental method to determine the impact of a particular intervention, it is important to note that a statistically significant difference in time trends was present in the pre-2015 period between the two groups (Tanzanians and refugees) and thus limits our ability to draw causal inference on the influx and surgical trends including the statistically significant difference-in-difference noted [21]. Nevertheless, this study still highlighted differential patterns of surgical utilization by two populations, including a statistically significant difference in difference of trends in surgery between refugees and Tanzanians in the pre- and post-2015 periods. Importantly, there appeared to be somewhat more missing data on nationality in the post-2015 era than the pre-2015 era and there appeared to be greater variability in number of surgeries across time among refugees in the post-2015 era. For the latter, we hypothesize this was for three reasons: (1) there was an expected general increase immediately following the influx of refugees; (2) there was an earthquake in 2017 during this time period that damaged the operating theatre; and (3) data from 2020 only included data through quarter 3. Similarly, the influx period used was January to March 2015 (2015, quarter 1), and Burundian refugees arrived beginning in April but over a period of a several months rather than a single month. Our use of quarterly time points somewhat addressed this concern, but the findings should be taken in this context. We have maintained using quarter 1 of 2015 so as to capture the large influx of persons from quarter 2. Lastly, some surgical cases cannot be managed at the dispensary level hospital and are thus referred outside of Nyarugusu refugee camp. These cases are not reflected in the surgical output characterized by this study, and thus our findings should not be misinterpreted as evidence of surgical need or burden of disease in this camp. Future research might consider exploring patterns and burden of surgical referral in similar contexts.

## Conclusions

Our study provides data on the differential patterns of surgical utilization among refugees and a host population in Tanzania. Analyzing a large dataset, we found that a significant portion of Tanzanians utilize the surgical services in the camp, and our data suggests that a large influx of refugees into Tanzania in 2015 seemingly did impact surgical utilization among the two sub-populations. These findings should be understood in light of the limitations above, but they do offer future avenues of research including additional time series models that may account for changing sub-population demographics, prospective registries, and studies seeking to better characterize compare prevalence estimates of the underlying burden of surgical disease in host and refugee communities.

## Data Availability

Data used and analyzed in the current study are not publicly available due to privacy and personally identifiable health information. De-identified, aggregate data is available from corresponding author upon request.
